# The Role of Inorganics in Preeclampsia Assessed by Multiscale Multimodal Characterization of Placentae

**DOI:** 10.3389/fmed.2022.857529

**Published:** 2022-03-30

**Authors:** Thomas Rduch, Elena Tsolaki, Yassir El Baz, Sebastian Leschka, Diana Born, Janis Kinkel, Alexandre H. C. Anthis, Tina Fischer, Wolfram Jochum, René Hornung, Alexander Gogos, Inge K. Herrmann

**Affiliations:** ^1^Laboratory for Particles Biology Interactions, Swiss Federal Laboratories for Materials Science and Technology (Empa), St. Gallen, Switzerland; ^2^Department of Gynaecology and Obstetrics, Kantonsspital St.Gallen, St. Gallen, Switzerland; ^3^Nanoparticle Systems Engineering Laboratory, Department of Mechanical and Process Engineering, Institute of Process Engineering, ETH Zürich, Zurich, Switzerland; ^4^Department of Radiology, Kantonsspital St.Gallen, St. Gallen, Switzerland; ^5^Institute of Pathology, Kantonsspital St.Gallen, St. Gallen, Switzerland

**Keywords:** calcifications, hypertension, electron microscopy, preeclampsia, elemental analysis (chemical), histology

## Abstract

Preeclampsia is one of the most dangerous diseases in pregnancy. Because of the hypertensive nature of preeclampsia, placental calcifications are believed to be a predictor for its occurrence, analogous to their role in cardiovascular diseases. However, the prevalence and the relevance of calcifications for the clinical outcome with respect to preeclampsia remains controversial. In addition, the role of other inorganic components present in the placental tissue in the development of preeclampsia has rarely been investigated. In this work, we therefore characterized inorganic constituents in placental tissue in groups of both normotensive and preeclamptic patients (*N* = 20 each) using a multi-scale and multi-modal approach. Examinations included elemental analysis (metallomics), sonography, computed tomography (CT), histology, scanning electron microscopy, X-ray fluorescence and energy dispersive X-ray spectroscopy. Our data show that tissue contents of several heavy metals (Al, Cd, Ni, Co, Mn, Pb, and As) were elevated whereas the Rb content was decreased in preeclamptic compared to normotensive placentae. However, the median mineral content (Ca, P, Mg, Na, K) was remarkably comparable between the two groups and CT showed lower calcified volumes and fewer crystalline deposits in preeclamptic placentae. Electron microscopy investigations revealed four distinct types of calcifications, all predominantly composed of calcium, phosphorus and oxygen with variable contents of magnesium in tissues of both maternal and fetal origin in both preeclamptic and normotensive placentae. In conclusion our study suggests that heavy metals, combined with other factors, can be associated with the development of preeclampsia, however, with no obvious correlation between calcifications and preeclampsia.

## Introduction

Preeclampsia (PE) is a severe pregnancy-associated disease and a major cause of morbidity and mortality affecting 3–8% of all pregnancies worldwide, accounting for over 50,000 deaths of mothers and their offspring annually (1). PE is characterized by hypertension (with systolic blood pressure ≥ 140 mmHg and diastolic blood pressure ≥ 90 mmHg) accompanied by proteinuria (≥ 300 mg/24 h) (2, 3). In clinics, a distinction is made between early onset and late-onset PE, with 34 weeks gestation being the cut-off value (4). An early sign of early onset PE is the abnormal placentation of cytotrophoblasts in the first trimester. In healthy pregnancies, the maternal spiral arterioles are re-modeled over time to allow a higher blood flow, accounting for the increasing nutrient demand (5). In early onset PE the spiral vessels do not complete the transformation from a proliferative epithelial to an invasive endothelial subtype, causing the diameters of the arteries to be narrower, subsequently leading to relative placental ischemia (6). Late onset PE is mainly induced through limited intervillous perfusion leading to chronic hypoxic placental tissue (7). Both early onset PE and late onset PE are associated with increased levels of proinflammatory and antiangiogenic factors in the maternal blood, including increased concentration of circulating soluble fms-like tyrosine kinase 1 (sFlt-1), which exacerbate the clinical symptoms of the mother (8).

The pathophysiology of PE and its manifestations in the placenta remain poorly understood. Research on PE has focused primarily on histoanatomical (9) and biochemical (5, 10) approaches, however, several studies indicate the potential importance of inorganics in the development of the disease, including macro and micro-nutrients, as well as (toxic) trace elements. For example, elevated concentrations of Cr, As, Pb, Ni, and Hg may contribute to the development of a proinflammatory and antiangiogenic environment by induction of oxidative stress, hence contributing to the development of PE (11–13). Most prior works have examined element concentrations of preeclamptic patients in the blood or urine. Studies analyzing preeclamptic placental tissue for elemental composition (major and/or trace elements) and minerals/calcifications, however, are relatively scarce (14, 15).

In addition to toxic trace elements, mineral deposits such as calcifications may play a key role in PE. Calcifications can be formed by metastatic, dystrophic or physiologic processes. Metastatic calcification is defined as the deposition of calcium in healthy tissue as a result of supersaturated calcium serum concentrations (16). Dystrophic calcification occurs in necrotic tissue while physiologic calcification is seen in bone and teeth formation (17, 18). The extent and tissue-specific patterns of vascular calcification are well-known predictors of cardiovascular morbidity and mortality. Indeed, placental calcifications similar to those found in aortic tissue have been reported recently (19). Despite the fact that PE is a hypertensive disease, placental calcifications are scarcely researched in this context. While calcifications are often observed in both healthy and diseased placentae, their relevance for clinical outcome remains controversial, in particular in PE (20–22). In a large study assessing placental histological sections of preeclampsia patients, Ezeigwe et al. found a correlation between placental infarcts and calcifications, however, failed to show a significant relationship between increased placental calcification and preeclampsia (23). In contrast, Grannum III calcifications were suggested to be a useful indicator of PE development later in the pregnancy (24). Furthermore, Chen et al. indicated that the presence of placental calcifications and weight gain during pregnancy might serve as a warning sign and requires closer surveillance for maternal and fetal well-being (25). Note, however, that most of the placental calcification research to date has focused on clinical sonography imaging and histological tissue analysis with poor sensitivity for mineral characteristics. A recent review by Wallingford et al. has stated that investigation is needed to delineate associations between preeclampsia, placental calcification, and vascular calcification in order to evaluate the potential diagnostic value of placental calcification in both acute and long-term cardiovascular health (26).

In this work, we therefore assess inorganic constituents in healthy and preeclamptic placentae with varying degrees of severity by conventional clinicophathological methods enriched by metallomics analysis and cutting-edge nano-analytical characterization. We present a route to augment the detection capabilities of histology by correlation with whole slide micro-X-ray fluorescence (μXRF) and backscattered electron (BSE) imaging in the scanning electron microscope (SEM), allowing the unambiguous identification and (chemical) characterization of placental solid inorganics (incl. calcifications) with nanometric resolution.

## Materials and Methods

### Patient Cohort and Placentae Harvesting

The study was approved by the Ethics Commission of Eastern Switzerland (EKOS 2020-01387). All placentae were collected directly after delivery at the Cantonal Hospital of St. Gallen, Switzerland, after obtaining informed consent from the patients. Placentae ranging from 25 + 6 weeks of pregnancy to 40 + 5 weeks of pregnancy were included. The corresponding control group included 20 term births between weeks 37 + 0 and 41 + 2 of gestation by healthy, normotensive women.

### Ultrasound Examination of the Placentae

Immediately after collection, the degree of placental calcification was assessed by an *ex vivo* ultrasound scan. The evaluation was carried out in analogy to the Grannum score which distinguishes between a smooth chronic plate (Grade 0), occasional parenchymal calcification (Grade I), deeper indentations of the chronic plate (Grade II) and significant basal plate calcification with indentations interrupting the chorionic plate (Grade III) (27). All ultrasound examinations were performed by a qualified obstetrician using a Voluson 730 (GE Medical Systems, Zipf, Austria) equipped with a transabdominal transducer operating in the frequency range between 2.8 and 10 MHz.

### Computed Tomography

Placentae were imaged within 1 h after delivery at the Department of Radiology Cantonal Hospital St. Gallen using a dual-source CT system (Somatom Force, Siemens Healthineers, Forchheim, Germany). Images were collected in single source mode using the following scanning parameters: tube voltage: 120 kilovoltage peak (kVp, peak potential applied to the x-ray tube, which accelerates electrons from the cathode to the anode); tube current-time product: 380 mAs; collimation; 192 × 0.6 mm; gantry rotation time; 250 ms; and pitch; 0.9. Tomography data were reconstructed using a slice thickness of 0.4 mm and an increment of 0.2 mm with a sharp convolution kernel (Ur77). Image data were reconstructed on a dedicated radiological workstation and evaluated by an experienced radiologist using commercially available CT analysis software (Extended Multi-planar Reconstruction plugin, impax ee, AGFA HealthCare, Mortsel, Belgium). In each CT dataset a threshold-based segmentation algorithm was used to quantify (i) the volume of the placenta by counting the volume of all voxels with a CT density between –150 and 150 Hounsfield units (HU), and (ii) the volume of calcified deposits by counting the volume of all voxels with a CT density of 180 HU or higher. The Hounsfield unit (sometimes also referred to as the “CT-unit”) is a relative measurement of the attenuation of X-rays by the specimen in computed tomography (CT) in relation to water and air, where water is arbitrarily set to 0 HU and air to –1,000 HU at standard temperature and pressure (28). In CT images, the Hounsfield scale is then displayed as gray tones.

### Elemental Analysis (Metallomics)

After ultrasound and CT imaging, five full thickness punch biopsies (8 mm diameter) were taken from each placenta in a circular pattern and stored in 70% ethanol. The total placental contents of major (Ca, P, Mg, Fe, K, and Na) and trace elements (Al, Cr, Mn, Co, Ni, Cu, Zn, As, Se, Rb, Sr, Ag, Cd, Cs, Ba, and Pb) were then determined. Trace elements were chosen according to previous reports with regard to PE [e.g., Cr, As, and Cd (29), Se (30), Sr (31), Pb (32)] as well as reported adverse effects on the placenta in general (33, 34) and their overall potential toxicity and abundance (e.g., Al, Cu, or Zn). A two-step tissue digestion procedure was used to avoid excessive foaming. First, the biopsies were dried for 24 h using a vacuum oven (SalvisLab, Renggli AG, Switzerland) typically yielding ≈ 50 mg in dry weight and then digested in 2.5 mL HNO_3_ in a pressurized microwave digestion system (MLS Turbowave, MLS GmbH, Leutkirch, Germany). Afterwards, 1 mL H_2_O_2_ was added to the samples and left to react for about 30 min until the samples were transparent and colorless. Digested samples were filled up to 50 mL with nanopure water and analyzed without further dilution using inductively coupled plasma optical emission spectroscopy (ICP-OES, Agilent 5110, Agilent Technologies Inc., CA, United States) for major elements and inductively coupled plasma mass spectroscopy (ICP-MS, Agilent 7900, Agilent Technologies Inc., CA, United States) for the trace elements. Measurements on the ICP MS were conducted using the high matrix introduction (HMI) mode and He as a collision cell gas for all analyzed elements, except As and Se, which were measured in high energy He mode. For quality assurance, the certified reference material BCR-185r (Bovine liver, European commission JRC, Institute for Reference Materials and Measurements, Belgium) with reference concentrations of 0.033 mg kg^–1^ As, 0.544 mg kg^–1^ Cd, 277 mg kg^–1^ Cu, 11.07 mg kg^–1^ Mn, 0.172 mg kg^–1^ Pb, 1.68 mg kg^–1^ Se and 138.6 mg kg^–1^ Zn was digested and analyzed with each batch of samples. Measured values for these elements in BCR-185r were well in agreement with the certified values (between 90 and 104% recovery, see Supplementary Table 2 for detailed element contents and element recoveries), supporting the suitability of the method.

### Histology

Tissue processing, preparation of histological sections and staining were performed using standard methods at the Institute of Pathology Cantonal Hospital of St. Gallen, Switzerland. The pathological examination included a macroscopic description of the location (central, paracentral and peripheral) of calcifications and targeted embedding followed by histological examination based hematoxylin eosin (HE) staining. The placentae were fixed with 4% buffered formalin and the relevant sampled areas were embedded in paraffin blocs. The blocs were cut into 2 μm sections, stained with HE and evaluated for pathological parameters using a conventional light microscope. In HE, calcified areas appear deep blue/purple.

In addition, Von Kossa staining was performed (35). To this end, unstained histological sections were deparaffinized by incubation in xylene for 10 min (2x) followed by rehydration in a decreasing ethanol gradient. After deparaffinization, the samples were incubated with 5% silver nitrate (abcam^®^ ab150687) for 45 min. Throughout the incubation time, the samples were exposed to a 60W incandescent light. Then, samples were rinsed with deionized water and incubated with 5% sodium thiosulfate for 3 min (abcam^®^ ab 150687). The samples were also counterstained with Nuclear Fast Red staining (abcam^®^ ab 150687) for tissue visualization. Finally, a coverslip was placed over the tissue using DPX mounting medium (Sigma-Aldrich 06522). After von Kossa staining, calcified areas appear black due to the reduced (metallic) silver.

### Micro-X-Ray Fluorescence Analysis (μXRF)

Unstained, deparaffinized histological sections were mounted on high purity vitreous carbon disks (Micro-to-nano, Netherlands) in order to allow for elemental analysis with virtually only C as a background element. Element distributions maps of the deparaffinized histological thin sections mounted on high purity vitreous carbon disks were recorded on a M4 Tornado micro X-ray fluorescence (μXRF) spectrometer (Bruker Nano GmbH, Berlin, Germany) equipped with an Ag cathode operated at an acceleration voltage of 50 kV. The spot size of the X-ray beam was 20 μm. Each map was scanned with a dwell time of 5 ms per pixel, with a total collection time of 6 h per map. Data were evaluated using Bruker software.

### Scanning Electron Microscopy

For SEM analysis, the carbon disks were then mounted on conventional aluminum stubs and coated with 10 nm carbon using a CCU-010 coater (Safematic, Switzerland). SEM imaging (secondary and backscattered electrons) and EDX analysis was performed on a Quanta 650 SEM (FEI, Thermo Fisher Scientific) at 10 kV. EDX Data was evaluated using Pathfinder 2.4 (Thermo Fisher Scientific). Large field of view stitch images of entire thin sections (secondary and backscattered electrons) were created on a Magellan 400 SEM (FEI, Thermo Fisher Scientific).

## Results

### Cohort Description

The entire study population was of Caucasian ethnicity (Table 1), with the majority of patients being over 30 years old. The prevalence of PE was higher in older patients, indicative of the increased likelihood of PE at higher age. All women had a singleton pregnancy and were mostly pregnant for the first time (N = 23). Most patients, namely 85% in the control and 80% in the PE group, were pre-obese (BMI 25–29.9) or obese (BMI > 30) at the time of delivery. In the PE-group, two patients admitted to smoke regularly during pregnancy.

**TABLE 1:** Characteristics of the study population.

	Control group (*n* = 20)	Preeclampsia group (*n* = 20)
**Age at sampling (years)**		
20–24	2	1
25–29	6	5
30–34	10	5
35–39	1	6
40–45	1	2
Mean	30.5	32.6
**Parity**		
1	9	14
2	6	4
3	4	0
4	0	1
5	1	1
Mean	1.9	1.55
**BMI at end of pregnancy**		
<18.5	0	0
18.6–24.9	3	4
25.0–29.9	10	6
>30.0	7	10
Mean	28.21	31.65
Smokers	0	2
Gestational age (average week)	39 + 4	35 + 6
Placenta weight in g (average)	505.2	383.5
**Grannum score**		
0	0	0
1	10	12
2	8	8
3	2	0
Mean	1.6	1.4

Based on the Grannum score, which is commonly used to assess placental calcifications during gestation, heavy calcifications (Grannum 3) were only found in two patients of the control group. The level of mild calcifications was very comparable between the groups. Thus, in this case the Grannum score was not indicative of PE development or the severity of PE. Not unexpectedly, the placenta weights were significantly lower in the PE compared to the normotensive group due to the preterm deliveries in the PE group (see Table 1).

### Metallomics Analysis of Bulk Tissue

**FIGURE 1:**
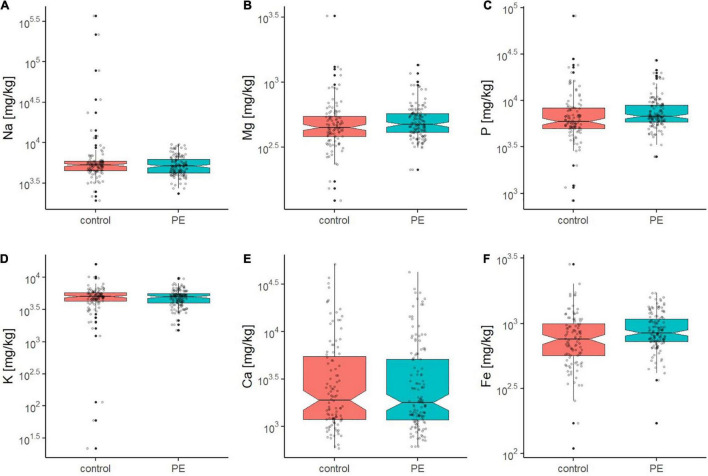
Boxplots **(A–F)** showing the concentration of Na, Mg, P, K, Ca, and Fe in the placental tissue of the control (red) and PE (green) placenta group as determined by ICP-OES. Outliers are displayed as filled black dots. The entire distribution of data points is shown as gray transparent dots. The black line displays the median and notches the 95% confidence interval of the median. For all displayed elements except Fe, control and PE group did not show significant differences in the mean (*p* > 0.05), also indicated by their overlapping notches.

**FIGURE 2:**
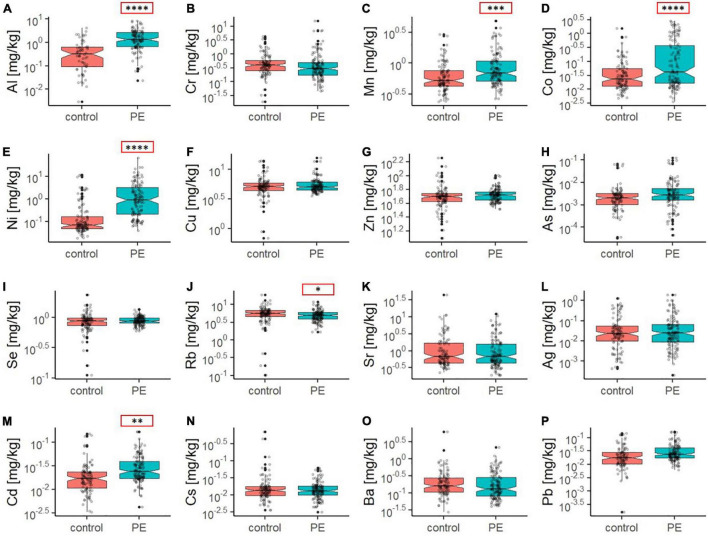
Boxplots **(A–P)** showing the concentration of trace elements in the placental tissue of the control (red) and PE (green) placenta group as determined by ICP-MS. Outliers are displayed as filled black dots. The entire distribution of data points is shown as gray transparent dots. The black line displays the median and notches the 95% confidence interval of the median. Note that non-overlapping notches strongly suggest a difference in the median. Additionally, asterisks in red boxes indicate significant differences in the mean element concentration between the control and PE group (with *p < 0.05, **p < 0.01, ***p < 0.001, and ****p < 0.0001, as determined by Welch two sample *t*-test).

In order to gain insights into the role of inorganics in healthy and preeclamptic placentae, bulk multi-element analysis was performed on placental tissue. Elemental concentrations in blood and urine may fluctuate during pregnancy due to physiological and metabolic changes (36). Thus, for a more robust survey, major elements (Figure 1) and trace elements (Figure 2) were analyzed directly in the placental tissue after postpartum by elemental analysis using ICP-MS (Supplementary Tables 1, 2).

In general, the median concentration levels of the major elements Mg, P, and Ca in placental tissue were very comparable to results reported in the literature (19) (Figure 1). No significant difference in Na, Mg, P, K, and Ca content between the control and PE group were found on a bulk level (Figures 1A–F). Only for Fe, significantly elevated levels were observed in the PE compared to the normotensive group (median values of 847 (PE) and 758 (control), Supplementary Table 2, ANOVA: *p* = 0.044).

Especially for Ca, the inter-subject variance was remarkably high with, however, comparable ranges in both groups. Calcium concentration ranged from 764 up to 21,597 mg/kg in the control and from 742 up to 19,496 mg/kg in the PE group (Supplementary Table 1), which corresponds to a 28 and 26 times higher maximum value compared to the minimum, respectively.

With regard to trace elements and heavy metals, levels of Al, Cr, Mn, Co, Ni, Cu, Zn, As, Se, Rb, Sr, Ag, Cd, Cs, Ba, and Pb were assessed in the placental tissues (Figure 2). One factor ANOVA indicates highly significant elevations in Al (p < 0.0001), Cd (*p* = 0.0012), Ni (*p* < 0.0001), Co (*p* < 0.0001) and Mn (*p* = 0.0003) as well as moderately higher Pb (*p* = 0.046) and As (*p* = 0.084) levels in the PE group on a bulk level. In contrast to the aforementioned elements, Rb showed a significantly lower concentration in the PE group (*p* = 0.034).

### Clinicopathological Analysis Enriched With Nano-Analytical Techniques

#### Computed Tomography

**FIGURE 3:**
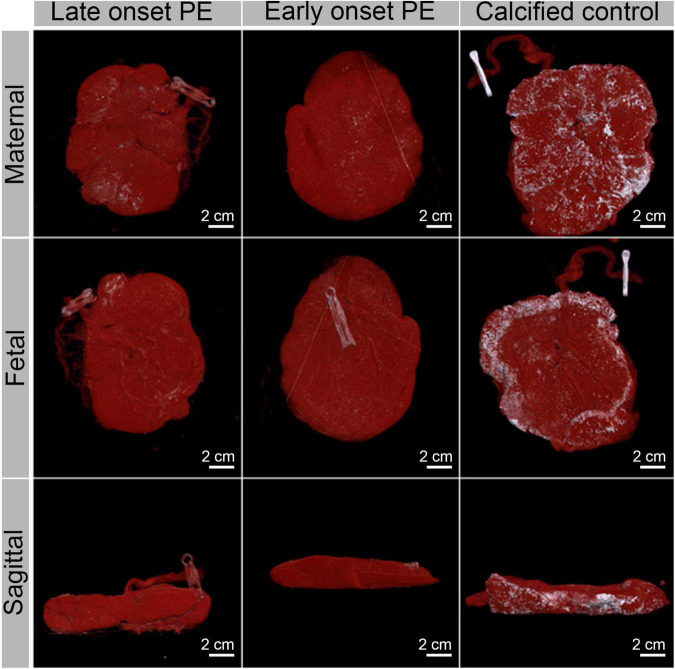
Computed tomography images of three representative placentae (a late onset PE placenta, an early onset PE placenta and a calcified control placenta, see Table 2 for their characteristics) in maternal, fetal and sagittal view. Soft tissue is shown in red while calcifications appear in white.

To assess macroscopic calcifications, all placentae were analyzed by CT. CT analysis allowed quantification of the macroscopic total calcified volume and extraction of information on the crystallinity/density of the calcifications based on the Hounsfield unit (HU) (see Supplementary Tables 3, 4 and Supplementary Figure 3). This analysis showed that both the calcified volume and the CT density of this volume (Ca HU) were significantly lower in the PE group (*p* = 0.02 and 0.0003, respectively). Representative early onset and a late onset placentae selected based on calcification content and clinical criteria (PE symptoms, Table 2) are displayed as reconstructed tomograms to illustrate the patterns of placental calcifications (Figure 3). Overall, mineral deposits in moderately calcified placentae (including both PE placentae) were predominantly found on the basal plate and in the periphery of the maternal side, with no apparent difference between normotensive and PE placentae. To illustrate the high variability in the extent of calcification, a heavily calcified placenta found in the control group was included in Figure 3. This placenta stemming from a clinically healthy mother (40 + 4 gestational weeks) showed extensive calcification, which reached from the basal plate up to the borders of the cotyledons, forming a cloud-like pattern.

**TABLE 2:** Characteristics of the three representative placentas selected for the multiscale imaging of calcific deposits.

	Control (calcified)	Early onset PE	Late onset PE
Maternal age	24	37	43
Gestational week	40 + 4	30 + 3	39 + 0
BMI start/end	17.3/23.1	20.4/26.4	27.5/30.4
Placental weight (g)	422	262	427
ASAT/ALAT	Not quantified	127/127 U/l	450/344 U/l
Platelets	137 G/l	135 G/l	28 G/l
Protein/creatinine ratio	Negative	2432.7 g/mol	60 g/mol
Smoker	No	No	No
Hounsfield Units (CT)	334.81	197.3	201.6
Ca (mg/kg)	4704.07	907.15	2453.84
P (mg/kg)	7144.57	5803.45	7942.66

#### Histology and Correlation With Nanoanalytical Characterization

**FIGURE 4:**
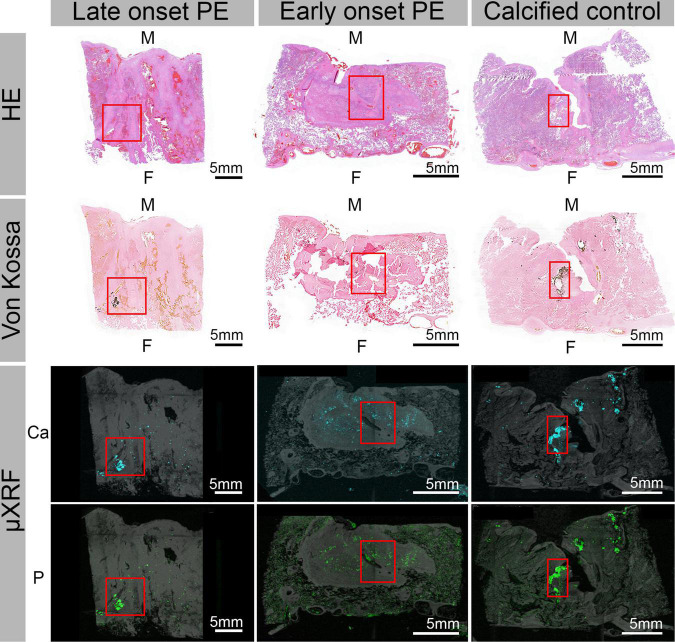
Hematoxylin eosin (HE) and von Kossa-stained histological sections as well as corresponding μ-X-ray fluorescence spectroscopy (μXRF) elemental maps for Ca and P obtained from three representative placentae. The μXRF maps were each overlayed with the corresponding camera image of the respective tissue for better visibility. From left to right: late onset PE, early onset PE and calcified control placenta. The letter M indicates the maternal and F the fetal side. Red boxes indicate highly calcified areas.

Following CT, we examined conventional HE- and von Kossa-stained histological sections collected from all the placentae. In agreement with the CT examination, most calcifications were located in the decidua on the maternal side of the placenta. Additional calcifications were also found in the intervillous space, in villi and in the periphery of the placentae, and in the central and paracentral part. Calcifications detectable in histology appeared either solitary, grouped or as large chunks. Selected placentae were additionally characterized with high-resolution techniques with chemical sensitivity, including micro X-ray fluorescence (μXRF) and whole slide scanning electron microscopy (WS-SEM). μXRF maps of placental tissue indicated calcium and phosphorus accumulations predominantly on the maternal side in the parenchyma (Figure 4). A reasonably good spatial correlation of the Ca and P detectable in μXRF with the visible calcifications in the HE and von Kossa staining was found (Figure 4). However, the spatial resolution of histology and μXRF imaging is limited to > 1μm, and hence no information on mineral morphology can be extracted. In contrast, WS-SEM enables the investigation of full histological slides with a spatial resolution of around 5 nm. By measuring back-scattered electrons, differences in atomic number (between soft tissue and minerals) can be assessed and mineral deposits can be readily identified as bright (electron-dense) spots. Due to the high spatial resolution of WS-SEM, mineral morphology can be assessed and correlated to histoanatomy.

**FIGURE 5:**
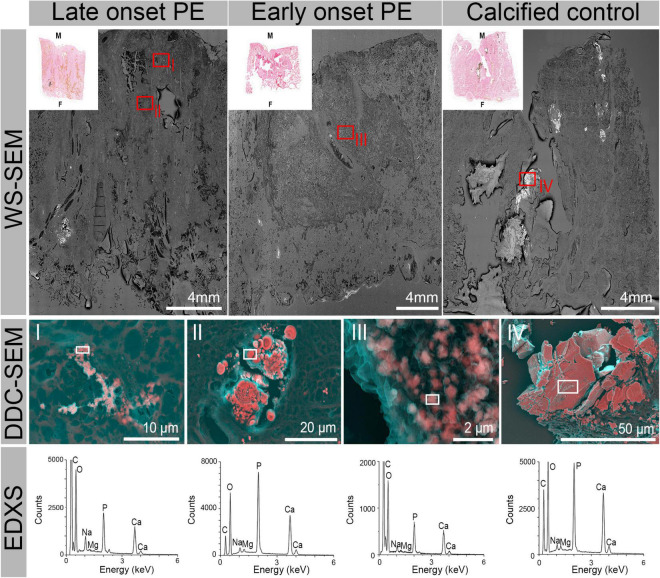
Whole slide scanning electron micrographs (WS-SEM) and corresponding HE histology micrographs from three representative placentae. From left to right: late onset PE, early onset PE and calcified control placenta. High magnification DDC-SEM illustrate the four characteristic subtypes of calcifications observed in the tissue samples: (I) needle like structures, (II) large spherical calcifications formed by concentric rings, (III) small spherical rough particles, (IV) large block calcifications of no specific internal structure (for higher detail images see also Supplementary Figure 1). Their localization is indicated by red boxes on the WS-SEM images. Energy-dispersive X-ray spectra (EDXS) indicate comparable composition of the particles (location of analysis indicated by white boxes), which mainly consist of Ca, P, and O with traces of Mg. Spectra obtained from surrounding non-calcified tissue support this statement (see Supplementary Figure 2). Carbon is part of the sample support and the tissue and hence also present in variable amounts.

**FIGURE 6:**
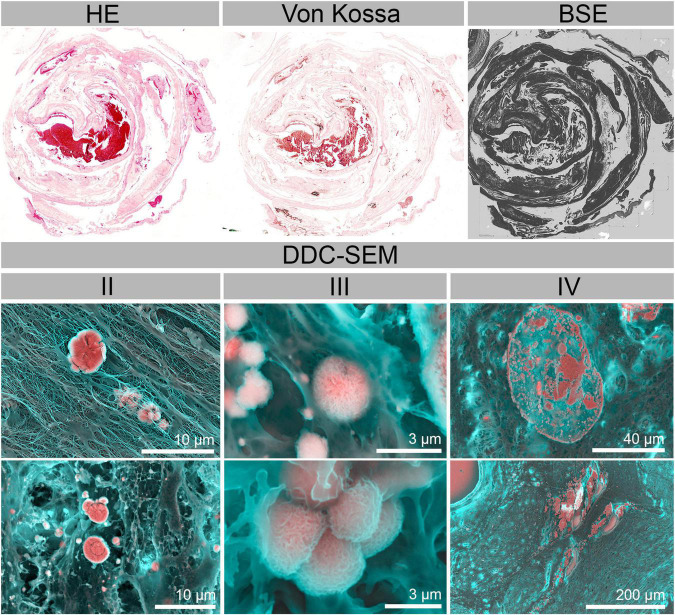
Hematoxylin eosin (HE) and von Kossa-stained histological section as well as a WS-SEM—BSE micrograph of a calcified decidua with amnion. Types of calcified particles found in selected regions [type II (large spherical calcifications), III (small spherical rough particles) and IV (large block calcifications)] are displayed in DDC-SEM micrographs.

Using WS-SEM and Density Dependent Color-SEM (DDC-SEM), four types of distinct morphologies of calcifications were identified (Figure 5 and Supplementary Figures 1, 2). These subtypes can be described as needle-like structures (Figure 5I), large spherical structures formed by concentric rings merged together forming larger minerals (Figure 5II), rough spherical particles (Figure 5III), which in some cases were also accumulating to form larger calcifications, and large calcification blocks of no distinct morphology (Figure 5IV). Again, and in line with CT and histological analyses, calcifications were found in the inter/intravillous space and the basal plate. Importantly, also much smaller deposits were readily detected and identified in WS-SEM, way beyond the CT detection limit. For example, micron-sized particles were found in the decidua beneath the subepithelial tissue of the amniotic membrane in portions of fetal tissue (Figure 6). Energy-dispersive X-ray spectroscopy showed that all types of calcifications were predominantly composed of Ca, P and O and contained Mg, albeit at variable amounts (Figure 5 and Supplementary Figure 2). X-ray emission related to N, S and Na stemmed mostly from surrounding/underlying tissue, whereas C originated from both tissue and sample support (Supplementary Figure 2).

## Discussion

Our analysis of major and trace elements in the placenta suggests that several metals (As, Cd, Ni, Pb, Al, Mn, and Co) are likely to be associated to the occurrence of PE as they were elevated in PE placentae. In the case of Rb, a deficiency in this element might be related to PE occurrence. Essential major elements (Ca, P, Mg, Na and K) and all other analyzed trace elements (Cr, Cu, Zn, Se, Sr, Ag, Cs, and Ba) were comparable in both groups. The comparable concentrations of Ca, P, and Mg in the two groups indicates a subordinate role of mineralization in PE, despite the disorder’s hypertensive origin. As total elemental analysis does not discriminate between particulate Ca, P and Mg and their freely dissolved, adsorbed or complexed states in the tissue, we further characterized the placentae using CT. Strikingly, both the total volume as well as the overall crystallinity/density of the calcifications were significantly higher in the control group. This indicates, that the development of calcifications in our study population was predominantly related to tissue aging rather than to the occurrence of PE.

Further nanoscale examinations of calcifications enabled identification of four distinct general types of calcifications [(I) needle-like structures, (II) large spherical calcifications formed by concentric rings, (III) small spherical rough particles, (IV) large block calcifications of no specific internal structure] which occurred in tissue of both normotensive and preeclamptic patients. In addition, the elemental composition of these deposits was very comparable between the study groups. Additionally, type III particles were found in both maternal and fetal tissue regions, including the decidua beneath the subepithelial connective tissue of the amnion. This gives an indication that not all calcifications in the placenta might be related to tissue aging.

Taken together, our results consistently suggest a subordinate role of calcifications in PE and pinpoint to a significant contribution of several trace elements and thus environmental factors to the development of PE.

While studies using bulk tissue analysis of the placenta are scarce, previous work on the role of several environmentally relevant metals in preeclampsia allows contextualization of our findings. Our data are in line with a previous study by Wang et al., which found that higher maternal blood concentrations of multiple metals, including Cr, As, Hg, and Pb, were associated with PE (11, 37). In particular, arsenic has previously been associated with adverse pregnancy outcomes, including low birth weight, anemia and spontaneous abortion (38). Also Cd is well-known to accumulate in the placenta (39). Higher levels of Cd have previously been also observed in placentae with parenchymal calcifications, regardless of smoking habit, gestational age, or birth weight (39). It is known that Cd can cause oxidative stress and may thus contribute to the development of PE (40). Indeed, increased placental tissue Cd levels have also been observed in PE cases in previous studies (14, 15). Cd levels in human placenta in studies between 1976 and 2011 ranged between 1.2 and 53 ng/g (39). Studies from 1978 to 1991 report concentrations of 2.6 up to 32 ng/g (39). Mean Cd concentration in placentae collected in TN, United States was 19 ng/g with a maximum value of 84 ng/g (41). Hence, these values are well in line with the ones observed in our study (median 6–30 ng/g control group and 10–83 ng/g PE group, Supplementary Table 1). Previous studies showed increased levels of Pb and Al in urine and serum of PE patients (32, 42, 43). Our results for the first time show that Al and Pb are also increased in placental tissue of PE patients. Pb and Al are known for inducing vasoconstriction and increasing the circulating levels of endothelin, which can induce PE (37, 42–44). Manganese plays a crucial part during the development of the nervous system and higher manganese concentration in maternal blood had previously been associated with lower risk of preeclampsia (44). Interestingly, in our cohort, Mn was higher in the PE group on a bulk level. Previous studies have indicated the strong concentration-dependent effect of Mn. Both deficiency in Mn as well as overexposure can increase reactive oxygen species generation and contribute to oxidative stress (45). With regard to Ni and Co, the concentrations were also elevated in the PE group compared to the normotensive control. There are few data regarding Ni and Co and their influence on preeclampsia. However, it is known that Ni can also cause oxidative stress and Co can cause pregnancy-associated hypertension, which can ultimately also lead to PE (46, 47). Rubidium as the only element that was reduced in the PE group has previously been associated with reduced fetal weight and pathological uterine Doppler (48).

Regarding distribution, mineral composition and ultrastructure of calcific deposits we found comparable results in both groups. Interestingly, spherical calcium phosphate deposits similar to those we detected in both maternal and fetal tissue were previously also found in osteocyte lacunae (49) and aortic tissue (16) and might thus be a physiological component of tissue.

The above findings suggest only weak trends of increased placental calcification in line with studies investigating calcifications in PE based on conventional clinicopathological studies (23, 50, 51). While several studies concentrated on ultrasound findings and correlated the amount of calcifications to the outcome of the pregnancy (20, 22) and drew a connection to increased stillbirth if the calcifications were seen before the 36th gestational week (20), these results cannot be compared to our study as they concentrate on calcifications in general and not in preeclampsia in particular. A recent review again showed associations between Grannum 3 calcifications and PE (52). Although the numbers of examined placentae in the reviewed studies is higher compared to this work, their results are based exclusively on the Grannum classification, which is known to have severe limitations, including observer bias (53). Also in our study, Grannum classification was not consistent with the quantitative data obtained from CT analysis (see Supplementary Figure 4). Thus, overall, we do not consider Grannum Score to be a suitable endpoint for assessing the extent of placental calcification for scientific purposes due to its aforementioned limitations. Sonographic 2D/3D imaging of the entire placenta may be a more suitable alternative. Alternatively, magnetic resonance imaging might offer additional insight and can also be performed during pregnancy (54), in sharp contrast to x-ray CT which is limited to postpartum analyses of the placenta due to radiation.

While the association of calcifications and PE remains controversial, and some studies suggest a positive correlation, we found no such association in our study. This could either be related to the significantly lower sample number, or a more direct analysis of calcifications by our materials characterization methods, making the quantification of minerals less prone to artifacts.

The good agreement with the available literature shows robustness of the metallomics approach in the bulk tissue and makes it a powerful route to profile lifestyle and environmental factors in pregnancy. The placenta as a whole, and especially also the metallomics fingerprint might serve as a time capsule containing highly relevant information on exposure to toxins and nutrient deficiencies encountered during pregnancy. Metallomics profiling thus offers a powerful and readily accessible route to investigate the implications (including life time risks) of such exposure in a comprehensive manner.

In addition to the bulk tissue analysis, we present a whole slide imaging approach with nanometric resolution to identify and characterize solid inorganic deposits in the placenta in their histoanatomical context with limits way beyond conventional histology. To the best of our knowledge, the present work is the first study to assess the ultrastructure and chemical composition of calcifications found in preeclampsia compared to normotensive placentae. In particular, WS-SEM with nanometric resolution and chemical sensitivity offers a powerful route to overcome the limitations of contemporary histology in assessing mineral deposits in the placenta but also a variety of other tissues prone to calcification. Using our multi-modal and multi-scale characterization approach, we are able to provide the full histoanatomical context to mineral structures, which in this case allowed us to directly compare calcific deposits found in inter/intravillous space regarding their distance to the basal plate (maternal side) and the amniotic (fetal) side and thus to assign them to either maternal or fetal-tissue origin. Further, this study demonstrated the presence of nanometer sized calcific deposits in every analyzed placental tissue that were not detectable by conventional histopathology.

While we have been able to confirm important differences in heavy metal contents, the small study population has an intrinsic risk for type II errors. Additionally, dietary habits and intake of dietary supplements may pose confounding factors, potentially being a root cause for increased concentration of Fe in the PE group. Also the fact that our control group represents healthy placentas at term while the study group includes diseased placentae from fetuses that were delivered prematurely poses a relevant yet unavoidable bias.

Additionally, the increased heavy metal concentrations found in placental tissue from preeclamptic patients are likely a direct consequence of increased exposure of the mother (55). However, we do not have complete anamnestic data on residence, working environment, passive smoking to conclusively show a correlation.

In summary, our metallomics analysis of normotensive and preeclamptic placentae is in agreement with the literature available for subsets of elements and demonstrates that heavy metals found elevated in blood and urine of PE patients are also detected at increasing levels in placental tissue, placing emphasis on their relation to the occurrence of the multifactorial disease. Thus, from a clinical perspective, the metallomics fingerprint analysis might serve as a time capsule containing highly relevant information on exposure to toxins and nutrient deficiencies encountered in pregnancy, offering a new route to early identification of patients at risk based on metallomics analyses of blood or urine during pregnancy. With regard to calcifications we show that, while no difference in Ca, P, and Mg was observed on a bulk level, the macroscopic particulate fraction was even lower in PE placentae which is an indication that placental age is the dominating factor leading to high particulate and crystalline Ca fractions. Additionally, our study adds further evidence for the limitations of the Grannum score, as we found a poor correlation between Grannum score and direct quantification of calcifications based on CT data (see Supplementary Figure 4).

In general, the methodology presented here may in future prove highly beneficial in accessing information stored in placental tissue and assessing its implications with regard to health later in life.

## Data Availability Statement

The original contributions presented in the study are included in the article/Supplementary Material, further inquiries can be directed to the corresponding author/s.

## Ethics Statement

The studies involving human participants were reviewed and approved by Ethics Commission of Eastern Switzerland (EKOS 2020-01387). The patients/participants provided their written informed consent to participate in this study.

## Author Contributions

TR was involved in the study design, collected, and analyzed clinical data. ET performed optical and electron microscopy studies and analyzed the data. YE and SL collected, analyzed, and interpreted radiological data. DB and WJ performed the histopathological analysis of placental tissue. AA developed the elemental analysis sample preparation method. JK and TF helped with sample and clinical data collection. RH contributed to the study design of the clinical study. AG developed analytical procedures for elemental and μXRF analyses. TR together with ET, AG and IH wrote the manuscript. IH conceived the study and supervised the work. All authors reviewed and approved the final version of the manuscript.

## Conflict of Interest

The authors declare that the research was conducted in the absence of any commercial or financial relationships that could be construed as a potential conflict of interest.

## Publisher’s Note

All claims expressed in this article are solely those of the authors and do not necessarily represent those of their affiliated organizations, or those of the publisher, the editors and the reviewers. Any product that may be evaluated in this article, or claim that may be made by its manufacturer, is not guaranteed or endorsed by the publisher.

## References

[1] Duley L. The global impact of pre-eclampsia and eclampsia. *Semin Perinatol.* (2009) 33:130–7. 10.1053/j.semperi.2009.02.010 19464502

[2] Phipps E Prasanna D Brima W Jim B. Preeclampsia: updates in pathogenesis, definitions, and guidelines. *Clin J Am Soc Nephrol.* (2016) 11:1102–13. 10.2215/CJN.12081115 27094609 PMC4891761

[3] American College of Obstetricians and Gynecologists, Task Force on Hypertension in Pregnancy. Hypertension in pregnancy. report of the American college of obstetricians and gynecologists’ task force on hypertension in pregnancy. *Obstet Gynecol.* (2013) 122:1122–31. 10.1097/01.AOG.0000437382.03963.88 24150027

[4] Robillard P-Y Dekker G Scioscia M Bonsante F Iacobelli S Boukerrou M Validation of the 34-week gestation as definition of late onset preeclampsia: testing different cutoffs from 30 to 37 weeks on a population-based cohort of 1700 preeclamptics. *Acta Obstet Gynecol Scand.* (2020) 99:1181–90. 10.1111/aogs.13846 32176317 PMC7492422

[5] Rana S Lemoine E Granger JP Karumanchi SA. Preeclampsia: pathophysiology, challenges, and perspectives. *Circ Res.* (2019) 124:1094–112. 10.1161/CIRCRESAHA.118.313276 30920918

[6] Brosens I Renaer M. On the pathogenesis of placental infarcts in pre-eclampsia. *J Obstet Gynaecol Br Commonw.* (1972) 79:794–9. 10.1111/j.1471-0528.1972.tb12922.x 4651288

[7] Khodzhaeva ZS Kogan YA Shmakov RG Klimenchenko NI Akatyeva AS Vavina OV Clinical and pathogenetic features of early- and late-onset pre-eclampsia. *J Matern Fetal Neonatal Med.* (2016) 29:2980–6. 10.3109/14767058.2015.1111332 26527472

[8] Palmer KR Tong S Kaitu’u-Lino TJ. Placental-specific sFLT-1: role in pre-eclamptic pathophysiology and its translational possibilities for clinical prediction and diagnosis. *Mol Hum Reprod.* (2017) 23:69–78. 10.1093/molehr/gaw077 27986932

[9] Roland CS Hu J Ren C-E Chen H Li J Varvoutis MS Morphological changes of placental syncytium and their implications for the pathogenesis of preeclampsia. *Cell Mol Life Sci.* (2016) 73:365–76. 10.1007/s00018-015-2069-x 26496726 PMC4846582

[10] Hansson SR Nääv Å Erlandsson L. Oxidative stress in preeclampsia and the role of free fetal he-moglobin. *Front Physiol.* (2015) 5:516. 10.3389/fphys.2014.00516 25628568 PMC4292435

[11] Wang Y Wang K Han T Zhang P Chen X Wu W Exposure to multiple metals and prevalence for preeclampsia in Taiyuan, China. *Environ Int.* (2020) 145:106098. 10.1016/j.envint.2020.106098 32916414

[12] Wu M-M Chiou H-Y Wang T-W Hsueh Y-M Wang I-H Chen C-J Association of blood arsenic levels with increased reactive oxidants and decreased antioxidant capacity in a human population of Northeastern Taiwan. *Environ Health Perspect.* (2001) 109:7. 10.1289/ehp.011091011 11675266 PMC1242077

[13] Vahter M. Effects of arsenic on maternal and fetal health. *Annu Rev Nutr.* (2009) 29:381–99. 10.1146/annurev-nutr-080508-141102 19575603

[14] Laine JE Ray P Bodnar W Cable PH Boggess K Offenbacher S Placental cadmium levels are associated with increased preeclampsia risk. *PLoS One.* (2015) 10:e0139341. 10.1371/journal.pone.0139341 26422011 PMC4589375

[15] Wang F Fan F Wang L Ye W Zhang Q Xie S. Maternal cadmium levels during pregnancy and the relationship with preeclampsia and fetal biometric parameters. *Biol Trace Elem Res.* (2018) 186:322–9. 10.1007/s12011-018-1312-3 29651732

[16] Bertazzo S Gentleman E Cloyd KL Chester AH Yacoub MH Stevens MM. Nano-analytical electron microscopy reveals fundamental insights into human cardiovascular tissue calcification. *Nat Mater.* (2013) 12:576–83. 10.1038/nmat3627 23603848 PMC5833942

[17] Karwowski W Naumnik B Szczepański M Myśliwiec M. The mechanism of vascular calcification. *Med Sci Monit.* (2012) 18:RA1–11. 22207127 10.12659/MSM.882181 PMC3560673

[18] Frink RJ Frink RJ. *Inflammatory Atherosclerosis.* Sacramento, CA: Heart Research Foundation (2002).

[19] Anthis AHC Tsolaki E Didierlaurent L Staubli S Zboray R Neels A Nano-analytical characterization of endogenous minerals in healthy placental tissue: mineral distribution, composition and ultrastructure. *Analyst.* (2019) 144:6850–7. 10.1039/C9AN01312A 31591608

[20] Chen K-H Seow K-M Chen L-R. The role of preterm placental calcification on assessing risks of stillbirth. *Placenta.* (2015) 36:1039–44. 10.1016/j.placenta.2015.06.015 26194801

[21] Chen KH Chen LR Lee YH. The role of preterm placental calcification in high-risk pregnancy as a predictor of poor uteroplacental blood flow and adverse pregnancy outcome. *Ultrasound Med Biol.* (2012) 38:1011–8. 10.1016/j.ultrasmedbio.2012.02.004 22475694

[22] Chen KH Chen LR Lee YH. Exploring the relationship between preterm placental calcification and adverse maternal and fetal outcome. *Ultrasound Obstet Gynecol.* (2011) 37:328–34. 10.1002/uog.7733 20586039

[23] Ezeigwe CO Okafor CI Eleje GU Udigwe GO Anyiam DC. Placental peripartum pathologies in women with preeclampsia and eclampsia. *Obstet Gynecol Int.* (2018) 2018:1–8. 10.1155/2018/9462938 30327674 PMC6171203

[24] McKenna D Tharmaratnam S Mahsud S Dornan J. Ultrasonic evidence of placental calcification at 36 weeks’ gestation: maternal and fetal outcomes. *Acta Obstet Gynecol Scand.* (2005) 84:7–10. 10.1111/j.0001-6349.2005.00563.x 15603560

[25] Chen K-H Seow K-M Chen L-R. Progression of gestational hypertension to pre-eclampsia: a co-hort study of 20,103 pregnancies. *Pregnancy Hypertens.* (2017) 10:230–7. 10.1016/j.preghy.2017.10.001 29153686

[26] Wallingford MC Benson C Chavkin NW Chin MT Frasch MG. Placental vascular calcification and cardiovascular health: it is time to determine how much of maternal and offspring health is written in stone. *Front Physiol.* (2018) 9:1044. 10.3389/fphys.2018.01044 30131710 PMC6090024

[27] Grannum PA Berkowitz RL Hobbins JC. The ultrasonic changes in the maturing placenta and their relation to fetal pulmonic maturity. *Am J Obstet Gynecol.* (1979) 133:915–22. 10.1016/0002-9378(79)90312-0 434036

[28] DenOtter TD Schubert J. *Hounsfield Unit.* Treasure Island, FL: StatPearls Publishing (2021). 31613501

[29] Rosen EM Muñoz MI McElrath T Cantonwine DE Ferguson KK. Environmental contaminants and preeclampsia: a systematic literature review. *J Toxicol Environ Health Part B.* (2018) 21:291–319. 10.1080/10937404.2018.1554515 30582407 PMC6374047

[30] Xu M Guo D Gu H Zhang L Lv S. Selenium and preeclampsia: a systematic review and meta-analysis. *Biol Trace Elem Res.* (2016) 171:283–92. 10.1007/s12011-015-0545-7 26516080

[31] Barneo-Caragol C Martínez-Morillo E Rodríguez-González S Lequerica-Fernández P Vega-Naredo I Álvarez Menéndez FV. Strontium and its role in preeclampsia. *J Trace Elem Med Biol.* (2018) 47:37–44. 10.1016/j.jtemb.2018.01.003 29544806

[32] Motawei SMH Attalla SM Gouda HE El-Harouny MA El-Mansoury AM. Lead level in pregnant women suffering from pre-eclampsia in Dakahlia, Egypt. *Int J Occup Environ Med.* (2013) 4:36–44. 23279796

[33] Punshon T Li Z Jackson BP Parks WT Romano M Conway D Placental metal concentrations in relation to placental growth, efficiency and birth weight. *Environ Int.* (2019) 126:533–42. 10.1016/j.envint.2019.01.063 30851484 PMC6475117

[34] Yin S Wang C Wei J Wang D Jin L Liu J Essential trace elements in placental tissue and risk for fetal neural tube defects. *Environ Int.* (2020) 139:105688. 10.1016/j.envint.2020.105688 32244100

[35] Puchtler H Meloan SN. Demonstration of phosphates in calcium deposits: a modification of von Kossa’s reaction. *Histochemistry.* (1978) 56:177–85. 10.1007/BF00495978 689915

[36] Gajewska K Błażewicz A Laskowska M Niziński P Dymara-Konopka W Komsta Ł. Chemical elements and preeclampsia – an overview of current problems, challenges and significance of recent research. *J Trace Elem Med Biol.* (2020) 59:126468. 10.1016/j.jtemb.2020.126468 32007824

[37] Sandoval-Carrillo A Méndez-Hernández EM Antuna-Salcido EI Salas-Pacheco SM Vázquez-Alaniz F Téllez-Valencia A Arsenic exposure and risk of preeclampsia in a Mexican mestizo population. *BMC Pregnancy Childbirth.* (2016) 16:153. 10.1186/s12884-016-0946-4 27401918 PMC4940694

[38] Naujokas MF Anderson B Ahsan H Aposhian HV Graziano JH Thompson C The broad scope of health effects from chronic arsenic exposure: update on a worldwide public health problem. *Environ Health Perspect.* (2013) 121:295–302. 10.1289/ehp.1205875 23458756 PMC3621177

[39] Esteban-Vasallo MD Aragonés N Pollan M López-Abente G Perez-Gomez B. Mercury, cadmium, and lead levels in human placenta: a systematic review. *Environ Health Perspect.* (2012) 120:1369–77. 10.1289/ehp.1204952 22591711 PMC3491942

[40] Chater S Douki T Garrel C Favier A Sakly M Abdelmelek H. Cadmium-induced oxidative stress and DNA damage in kidney of pregnant female rats. *C R Biol.* (2008) 331:426–32. 10.1016/j.crvi.2008.03.009 18510995

[41] Mikelson CK Troisi J LaLonde A Symes SJK Thurston SW DiRe LM Placental concentrations of essential, toxic, and understudied metals and relationships with birth outcomes in Chattanooga, TN. *Environ Res.* (2019) 168:118–29. 10.1016/j.envres.2018.09.006 30296639 PMC6288679

[42] Elongi Moyene J-P Scheers H Tandu-Umba B Haufroid V Buassa-bu-Tsumbu B Verdonck F Preeclampsia and toxic metals: a case-control study in Kinshasa, DR Congo. *Environ Health.* (2016) 15:48. 10.1186/s12940-016-0132-1 27044488 PMC4820935

[43] Maduray K Moodley J Soobramoney C Moodley R Naicker T. Elemental analysis of serum and hair from pre-eclamptic South African women. *J Trace Elem Med Biol.* (2017) 43:180–6. 10.1016/j.jtemb.2017.03.004 28325649

[44] Liu T Hivert M-F Rifas-Shiman SL Rahman ML Oken E Cardenas A Prospective as-sociation between manganese in early pregnancy and the risk of preeclampsia. *Epidemiology.* (2020) 31:677–80. 10.1097/EDE.0000000000001227 32618710 PMC7398820

[45] Li L Yang X. The essential element manganese, oxidative stress, and metabolic diseases: links and interactions. *Oxid Med Cell Longev.* (2018) 2018:e7580707. 10.1155/2018/7580707 29849912 PMC5907490

[46] Viemann D Schmidt M Tenbrock K Schmid S Müller V Klimmek K The contact allergen nickel triggers a unique inflammatory and proangiogenic gene expres-sion pattern via activation of NF-κB and hypoxia-inducible factor-1α. *J Immunol.* (2007) 178:3198–207. 10.4049/jimmunol.178.5.3198 17312168

[47] Liang C Wang J Xia X Wang Q Li Z Tao R Serum cobalt status during pregnancy and the risks of pregnancy-induced hypertension syndrome: a prospective birth cohort study. *J Trace Elem Med Biol.* (2018) 46:39–45. 10.1016/j.jtemb.2017.11.009 29413109

[48] Gómez-Roig M Mazarico E Cuadras D Muniesa M Pascal R Ferrer P Placental chemical elements concentration in small fetuses and its relation-ship with Doppler markers of placental function. *Placenta.* (2021) 110:1–8. 10.1016/j.placenta.2021.05.001 34051643

[49] Milovanovic P Zimmermann EA vom Scheidt A Hoffmann B Sarau G Yorgan T The formation of calcified nanospherites during micropetrosis represents a unique mineralization mechanism in aged human bone. *Small.* (2017) 13:1602215. 10.1002/smll.201602215 28084694

[50] Correia-Branco A Rincon MP Pereira LM Wallingford MC. Inorganic phosphate in the pathogenesis of pregnancy-related complications. *Int J Mol Sci.* (2020) 21:5283. 10.3390/ijms21155283 32722465 PMC7432618

[51] Narasimha A Vasudeva DS. Spectrum of changes in placenta in toxemia of pregnancy. *Indian J Pathol Microbiol.* (2011) 54:15. 10.4103/0377-4929.77317 21393870

[52] Schiffer V van Haren A De Cubber L Bons J Coumans A van Kuijk SM Ultrasound evaluation of the placenta in healthy and placental syndrome pregnancies: a systematic review. *Eur J Obstet Gynecol Reprod Biol.* (2021) 262:45–56. 10.1016/j.ejogrb.2021.04.042 33984727

[53] Cooley SM Donnelly JC Walsh T McMahon C Gillan J Geary MP. The impact of ultrasonographic placental architecture on antenatal course, labor and delivery in a low-risk primigravid popula-tion. *J Matern Fetal Neonatal Med.* (2011) 24:493–7. 10.3109/14767058.2010.497877 20608801

[54] Pietsch M Ho A Bardanzellu A Zeidan AMA Chappell LC Rutherford M APPLAUSE: automatic prediction of PLAcental health via U-net Segmentation and statistical evaluation. *Med Image Anal.* (2021) 72:102145. 10.1016/j.media.2021.102145 34229190 PMC8350147

[55] Pigatto PD Minoia C Ronchi A Guzzi G. Human placenta and markers of heavy metals exposure. *Environ Health Perspect.* (2013) 121:a10. 10.1289/ehp.1206061 23287423 PMC3553444

