# Supplementary material for: The Role of Inorganics in Preeclampsia Assessed by Multiscale Multimodal Characterization of Placentae
Source: Front Med (Lausanne). 2022 Mar 30;9:857529. doi: 10.3389/fmed.2022.857529 (PMC9009444; doi:10.3389/fmed.2022.857529)
Supplement: Supplementary file 1 [file Data_Sheet_1.pdf]

SUPPLEMENTAL INFORMATION TO: The Role of Inorganics in Preeclampsia Assessed by Multiscale Multimodal Characterization of Placentae

***AUTHORS**: Thomas RDUCH[a,b],MD ; Elena TSOLAKI[a,c],PhD;Yassir EL BAZ[d], MD; Sebastian LESCHKA[d], MD; Diana BORN [e],MD; Janis KINKEL[b], MD; Alexandre H.C. ANTHIS[a,c], PhD Tina FISCHER[b],*MD; *Wolfram JOCHUM[e],MD; René HORNUNG[b*],MD; Alexander GOGOS[a,c*],PhD and Inge K. HERRMANN[a,c*], PhD*

AFFILIATIONS:

[a]Laboratory for Particles Biology Interactions, Swiss Federal Laboratories for Materials Science and Technology (Empa), Lerchenfeldstrasse 5, CH-9014, St. Gallen, Switzerland.

[b]Department of Gynaecology and Obstetrics, Cantonal Hospital St. Gallen (KSSG), Rorschacherstrasse 95, CH-9007 St. Gallen, Switzerland.

[c]Nanoparticle Systems Engineering Laboratory, Institute of Process Engineering, Department of Mechanical and Process Engineering, ETH Zurich, Sonneggstrasse 3, CH-8092 Zurich, Switzerland.

[d]Department of Radiology, Cantonal Hospital St.Gallen (KSSG), Rorschacherstrasse 95, CH-9007 St.Gallen, Switzerland

[e]Institute of Pathology, Cantonal Hospital St.Gallen (KSSG), Rorschacherstrasse 95, CH-9007 St.Gallen, Switzerland

SUPPLEMENTAL FIGURES

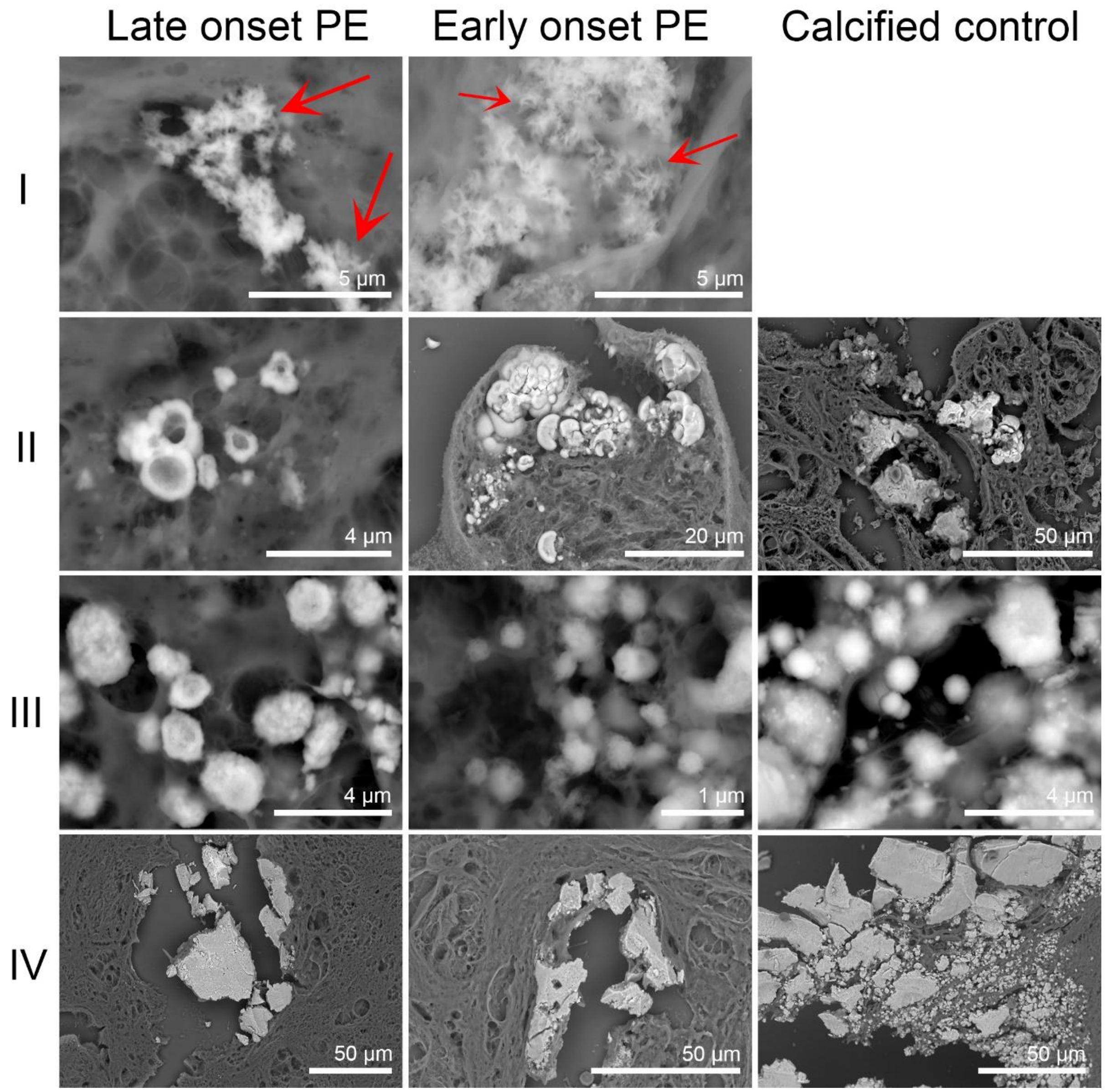

Supplemental Information Figure 1: Types of calcifications observed in selected plaecentae

Backscatter electron micrographs of the different subtypes of calcifications observed in the tissue samples. The needle like structures (red arrows) were absent in the calcified control.

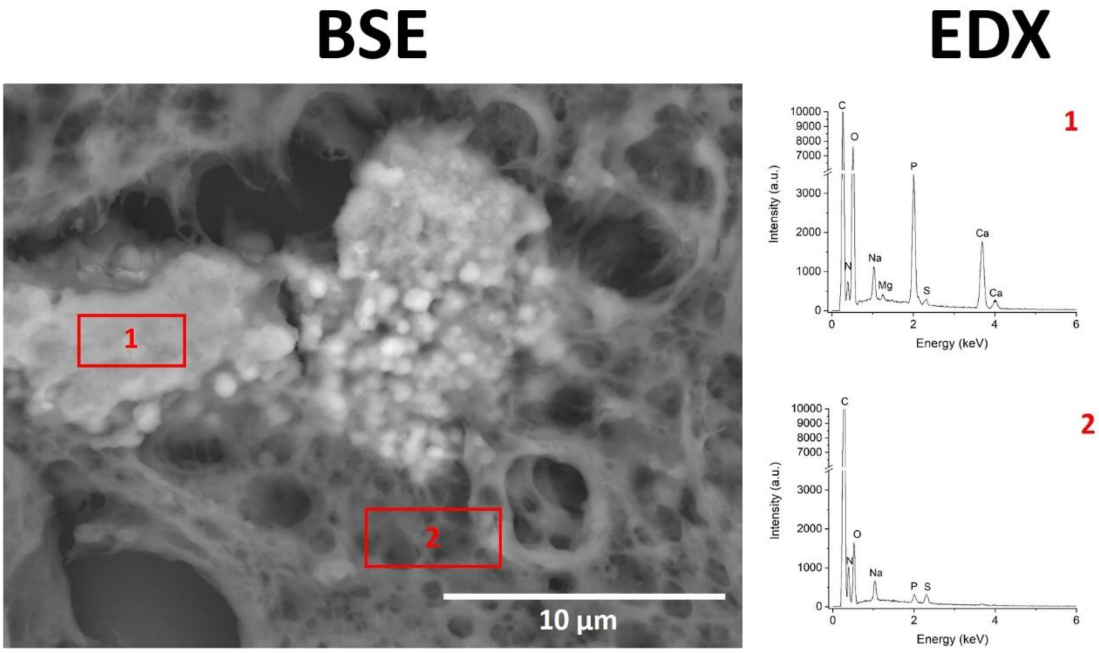

Supplemental Information Figure 2: Comparison of elemental signatures from a calcific deposit and its surrounding tissue

Comparison of EDX spectra typically obtained from a calcific deposits (1) and from sur-rounding tissue areas (2). This particular image was obtained from the selected late onset PE placenta (Main manuscript Table 2).

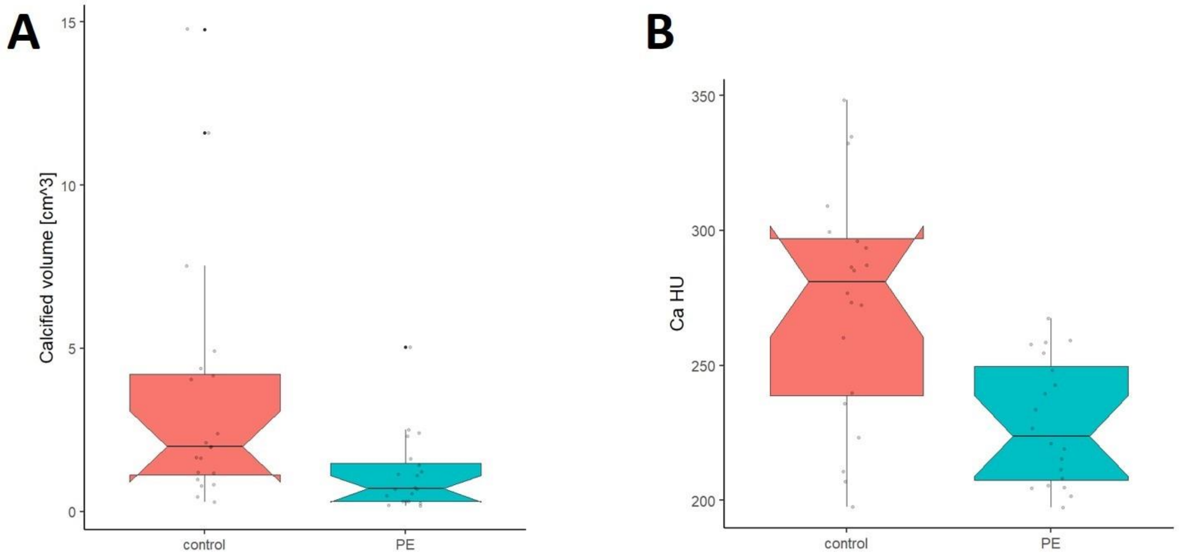

Supplemental Information Figure 3: Volume and associated Hounsfield units of calcifications in the analyzed placentae as determined by CT analysis.

Calcified volume in cm^3 (A) and CT density of the calcifications in Hounsfield units (Ca HU) (B) of PE and normotensive placentae.

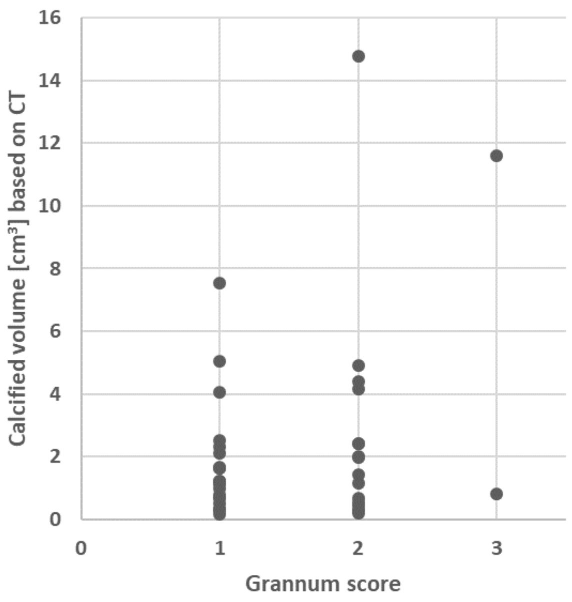

Supplemental Information Figure 4: Grannum score in relation to the calcified volume as determined by CT of all analyzed placentae.

Calcified volume in cm^3 (determined from CT analysis) vs Grannum score of PE and normo-tensive placentae.

SUPPLEMENTAL TABLES

Supplemental Information Table 1: Ranges of median placental element concentrations in µg g^{-1} from normotensive (control) and preeclamptic patients (PE).

	Control		PE	
**Element**	**Min**	**Max**	**Min**	**Max**
**Ca**	764	21597	742	19496
**P**	3540	14300	4323	17215
**Mg**	312	745	357	885
**Na**	3314	77920	3096	8433
**Fe**	481	1648	532	1345
**K**	115	6263	3009	6276
**Al**	0.038	1.25	0.16	4.26
**Cr**	0.042	1.29	0.065	1.44
**Mn**	0.26	1.06	0.36	2.08
**Co**	0.010	0.23	0.005	2.10
**Ni**	0.041	2.43	0.074	6.55
**Cu**	3.49	7.31	4.22	8.58
**Zn**	36.69	57.09	39.87	71.99
**As**	0.0004	0.006	0.0003	0.016
**Se**	0.50	1.10	0.74	1.15
**Rb**	0.42	8.90	3.11	8.61
**Sr**	0.37	4.71	0.31	7.15
**Ag**	0.001	0.43	0.002	0.44
**Cd**	0.006	0.031	0.010	0.083
**Cs**	0.005	0.13	0.005	0.051
**Ba**	0.051	0.72	0.046	0.77
**Pb**	0.004	0.063	0.012	0.054

Supplemental Information Table 2: Major and trace element contents (µg g^{-1}) in control (N=20) and PE placentas (N=20) as well as values determined for the certifier reference material BCR185R (Bovine liver). Analytical recovery is calculated as $R_c\% = \frac{\text{mean measured concentration}}{\text{certified element concentration (BCR−185R)}} \cdot 100$

	Mean			Median			Standard deviation			Analytical recovery %
**Element**	**Control**	**PE**	**BCR-185R**	**Control**	**PE**	**BCR-185R**	**Control**	**PE**	**BCR-185R**	**BCR-185R**
**Ca**	5308	4605	179	1895	1791	159	8277	6646	63	n.a.
**P**	8040	7943	13511	5947	6737	13119	8572	3839	2268	n.a.
**Mg**	518	518	720	450	474	686	335	173	147	n.a.
**Na**	11975	5359	2494	5266	5112	2373	41584	1568	486	n.a.
**Fe**	825	916	209	758	847	194	407	291	41	n.a.
**K**	5039	4894	12776	5026	4986	12162	2026	1455	2457	n.a.
**Al**	0.61	1.90	0.97	0.32	1.35	0.71	0.82	1.69	0.92	n.a.
**Cr**	0.63	0.74	0.47	0.40	0.30	0.39	0.74	1.67	0.29	n.a.
**Mn**	0.68	0.98	11.44	0.52	0.69	10.98	0.46	0.80	1.65	103
**Co**	0.08	0.38	0.31	0.02	0.04	0.27	0.18	0.64	0.08	n.a.
**Ni**	0.60	3.10	0.38	0.07	0.89	0.17	1.84	6.93	0.50	n.a.
**Cu**	5.35	5.54	287.07	5.15	5.02	266.78	2.04	1.82	50.29	104
**Zn**	51.71	53.42	130.37	49.78	52.78	121.44	21.80	11.91	21.61	94
**As**	0.004	0.008	0.029	0.002	0.003	0.029	0.011	0.019	0.004	89
**Se**	0.88	0.90	1.58	0.88	0.88	1.52	0.29	0.14	0.24	94
**Rb**	5.73	5.11	20.00	5.67	4.98	19.45	2.53	1.84	2.57	n.a.
**Sr**	1.97	1.46	0.11	0.68	0.67	0.09	4.75	1.99	0.05	n.a.
**Ag**	0.10	0.12	0.14	0.02	0.02	0.06	0.20	0.29	0.16	n.a.
**Cd**	0.02	0.03	0.56	0.02	0.02	0.54	0.02	0.03	0.08	103
**Cs**	0.028	0.017	0.023	0.014	0.013	0.022	0.080	0.011	0.003	n.a.
**Ba**	0.31	0.24	0.04	0.16	0.13	0.03	0.64	0.27	0.03	n.a.
**Pb**	0.03	0.03	0.18	0.02	0.02	0.17	0.03	0.03	0.03	102

Supplemental Information Table 3: Computed tomography data set of control placentae.

Number	Volume [cm^3]	HU avg	HU stdev	Ca vol [cm^3]	Ca HU	Age [y]	Week
**1**	638.52	32.86	100.24	11.61	273.31	26	40.85
**2**	410.96	26.46	89.07	0.99	276.91	28	38.85
**3**	440.77	31.41	86.26	7.53	299.56	34	40.42
**4**	336.22	27.86	94.74	1.66	348.29	34	38.42
**5**	496.02	33.2	87.51	1.21	260.32	26	40
**6**	602.61	30.98	90.19	4.06	293.61	26	40
**7**	393.92	32.55	87.2	2	285.29	30	40
**8**	474.08	32.91	82.31	0.44	235.81	32	39
**9**	374.77	23.02	101.35	0.82	239.82	33	37.28
**10**	471.26	33.24	84.73	0.78	207.05	37	38.28
**11**	520.8	23.04	90.93	1.64	197.72	31	39.28
**12**	422.05	28.38	96.55	1.17	223.28	26	39.57
**13**	686.67	15	117.64	4.92	286.45	34	40.28
**14**	698.85	28.56	97.55	4.38	287.2	33	40.28
**15**	553.95	27.35	89.08	2.4	272.41	23	39.14
**16**	591.08	36.21	108.66	14.78	334.81	24	40.57
**17**	410.33	23.13	93.14	1.98	332.38	42	40.28
**18**	439.94	30.47	91.6	2.12	296.1	25	38.28
**19**	436.05	27.86	85.6	0.29	210.65	33	41.28
**20**	353.2	19.82	114.49	4.16	309.03	33	39.28

Supplemental Information Table 4: Computed tomography data set of preeclamptic placentae

Number	Volume [cm^3]	HU avg	HU stdev	Ca vol [cm^3]	Ca HU	Age [y]	Week
**1**	559.7	30.95	88.94	1.62	254.59	29	40.42
**2**	390.87	6.51	133.69	0.68	204.66	35	38.14
**3**	363.93	7.17	129.32	0.54	205.46	33	34.57
**4**	208.88	8.95	127.28	0.32	208.3	22	31.42
**5**	268.19	23.75	103.32	0.3	197.3	37	30.42
**6**	325.29	2.2	135.81	0.49	211.42	36	33.28
**7**	348.24	14.88	118.87	1.14	267.42	31	38
**8**	473.01	19.79	110.86	2.51	201.6	43	39
**9**	423.68	10.79	116.05	0.31	215.38	28	34.71
**10**	232.29	26.74	100.5	1.42	259.32	40	37
**11**	350.73	26.76	94.05	1.22	226.7	33	35
**12**	251.68	24.85	99.12	0.2	221.08	29	25.85
**13**	521.9	17.08	98.82	0.26	242.64	40	35.42
**14**	580	22.49	103.97	2.31	239.69	36	39.28
**15**	226.98	16.15	100.31	0.17	204.6	29	30.85
**16**	468.9	30.62	84.73	0.72	258.42	31	37.42
**17**	595.28	13.75	118.24	1.1	219.14	35	40.71
**18**	342.28	13.57	117.12	2.41	257.93	36	35.57
**19**	379.7	6.91	128.82	5.04	233.75	25	38.42
**20**	445.83	10.63	106.6	0.68	248.11	25	40.57
